# Monitoring the Invasion of *Spartina alterniflora* from 1993 to 2014 with Landsat TM and SPOT 6 Satellite Data in Yueqing Bay, China

**DOI:** 10.1371/journal.pone.0135538

**Published:** 2015-08-11

**Authors:** Anqi Wang, Jiadai Chen, Changwei Jing, Guanqiong Ye, Jiaping Wu, Zhixing Huang, Chaosheng Zhou

**Affiliations:** 1 Institute of Islands and Coastal Ecosystems, Zhejiang University, Hangzhou, China; 2 Zhejiang Mariculture Research Institute, Wenzhou, China; Louisiana State University Agricultural Center, UNITED STATES

## Abstract

The exotic plant *Spartina alterniflora* was introduced to Yueqing Bay more than 20 years ago for tidal land reclamation and as a defense against typhoons, but it has rapidly expanded and caused enormous ecological consequences. Mapping the spread and distribution of *S*. *alterniflora* is the first step toward understanding the factors that determine the population expansion patterns. Remote sensing is a promising tool to monitor the expansion of *S*. *alterniflora*. Twelve Landsat TM images and Support Vector Machine (SVM) were used to delineate the invasion of *S*. *alterniflora* from 1993 to 2009, and SPOT 6 images and Object-Based Image Analysis (OBIA) were used to map the distribution of *S*. *alterniflora* in 2014. In situ data and Unmanned Aerial Vehicle (UAV) images were used as supplementary data. *S*. *alterniflora* spread rapidly in Yueqing Bay over the past 21 years. Between 1993 and 2009, the area of *S*. *alterniflora* increased by 608 times (from 4 to 2432 ha). The rapid expansion of *S*. *alterniflora* covered almost all of the bare mudflats around the mangrove forests and the cultivated mudflats. However, from 2009 to 2014, the rate of expansion of *S*. *alterniflora* began to slow down in Yueqing Bay, and the total area of *S*. *alterniflora* in Yantian decreased by 275 ha. These phenomena can be explained by the landscape changes and ecological niches. Through the expansion of *S*. *alterniflora*, it was found that the ecological significance and environmental impact of *S*. *alterniflora* was different in different regions in Yueqing Bay. The conservation plans for Yueqing Bay should consider both the positive and negative effects of *S*. *alterniflora*, and the governmental policy should be based on the different circumstances of the regions.

## Introduction

Biological invasions have become one of the main components of global change over the past 50 years and have adversely affected ecosystem functioning and biodiversity [1–3]. In particular, invasive plants can pose a serious threat to local ecological environments, cause appreciable losses to the regional economy and change the local vegetation type, soil properties, biogeochemical cycles, patterns of herbivory, and disturbance regimes [4–6]. *Spartina alterniflora*, which is a perennial rhizomatous C4 grass of Phocaea, is a common salt marsh vegetation that is native to the intertidal zones on the Atlantic and Gulf of Mexico coastal areas of North America. It was intentionally introduced to China in December 1979 [7]. Indeed, the original purposes of introducing it were for seashore stabilization, tidal land reclamation, and soil improvement. It has several biological traits that make it a common species for ecological restoration, such as rapid growth, high productivity, a well-developed subsurface system, and high tolerance to salt [6,8–12]. It then however soon became an invasive species. In the early 1990s, *S*. *alterniflora* began to expand rapidly in the intertidal zone. With this expansion, it has become the main exotic plant along the Chinese coast, and the area of *S*. *alterniflora* reached 34,451 ha in 2007 [13]. In recent years, several studies have indicated that the rapid expansion of *S*. *alterniflora* may threaten the native mangrove ecosystems and mudflat cultivation environments has negative impacts on sediment transport, accumulation, and wetland material cycling and causes declines in biodiversity [13–17]. It was therefore written on the top list of 16 invasive species issued by the State Environmental Protection Administration of China in 2003 [13].

As early as 1983, Zhejiang Province was first introduced 16 m^2^ of *S*. *alterniflora* in the Wumen mudflat of Tongli, Yuhuan County [7]. In 2009, after more than 20 years of invasion, *S*. *alterniflora* covered 5092 ha in Yueqing Bay, which is the largest salt marsh in Zhejiang Province [7]. Previous research showed that the introduction of *S*. *alterniflora* to Yueqing Bay was beneficial to coastal protection and tidal land reclamation [7,9,13,18]. However, the mangrove ecosystems and mudflat cultivation environment in the national conservation zone have also been threatened by the rapid expansion of *S*. *alterniflora*. There is a growing need to monitor the spread and determine the distribution of *S*. *alterniflora* to support comprehensive prevention and control measures and to conserve the local ecological environment. The detailed and up-to-date maps of the invasion of *S*. *alterniflora* are lacking in Yueqing Bay.

Remote sensing is a tool that provides viable methods to map invasive plants and monitor vegetation dynamics across broad geographic extents [19]. Landsat TM images with 30-m resolution have been extensively used for ecosystem monitoring, and the almost 40-year-long record of Landsat imagery provides a rich dataset that can facilitate mapping *S*. *alterniflora* [12,20,21]. However, images from TM satellite sensor are insufficient for more detailed maps of invasive species spread [21], because of the relatively low resolution of moderate spatial satellite imagery for determining an accurate distribution of *S*. *alterniflora*. The availability of new global observation datasets, such as SPOT 6 images, which are acquired at a spatial resolution of 6 meters and a panchromatic band of 1.5 meters and have a higher spatial resolution than SPOT 5, has revolutionized the utility of contemporary methods for mapping the distribution of *S*. *alterniflora* [22]. The limitation of using SPOT 6 images is their high cost and the extremely lack of historic data. We used both TM and SPOT 6 images in this study to delineate the expansion and map the distribution of *S*. *alterniflora*.

Coastal land cover change classes obtained from multi-temporal datasets are often characterized by complex class distributions (multi-modal, non-normal). In previous studies, for example, in Jiangsu and the Yangtze River Estuary, Shanghai, Landsat TM images and a supervised classification, the Maximum Likelihood Classifier (MLC) was used to extract *S*. *alterniflora*, but the initial classification accuracy was not very high (60% to 80%) [12,20]. SVM, which is a non-parametric classifier, is therefore better suited for change classifications than parametric classification algorithms (e.g. maximum likelihood) [23] and has successfully been combined with change detection techniques using Landsat TM images to map biological invasions [21]. OBIA is widely used to classify high resolution images, in Dongtan of Chongming Island, China, they used the high resolution image and OBIA for mapping plants in a saltmarsh ecosystem [24], it was easy to understand that high spatial resolution images resulted in more detailed imagery, but it was also worth noting that land use classification accuracy may decrease due to the increased intra-class variability inherent from more detailed and higher resolution data [25], as traditional classifications were designed for pixel-based methods, the “salt-and-pepper” problem of traditional classifications become more serious when the spatial resolution of satellite images increased, which were not suitable for processing of high resolution images due to lack of spatial information [24]. However, OBIA performs the segmentation of groups of image pixels into a smaller number of homogenous image objects based on both spectral and spatial information [26,27]. OBIA can extract real world objects with both high classification accuracy and proper shapes, which is difficult to accomplish using traditional classification methods [28]. In this research, Support Vector Machine (SVM) and Object-Based Image Analysis (OBIA) were used for the interpretation.

The spread of *S*. *alterniflora* and its current status in Yueqing Bay can be determined through the analysis of the satellite images. While previous researches has demonstrated the potential for mapping *S*. *alterniflora* from Landsat TM/ETM+ images or other high spatial resolution satellite images [12,20,24], no previous research has assessed *S*. *alterniflora* expansion by SPOT 6 images. Hence, the *S*. *alterniflora* invasion provides an opportunity to test the use of SVM and OBIA as classifiers for the detailed monitoring of *S*. *alterniflora* invasions using a time series of Landsat TM images and high spatial resolution images (SPOT 6). The objectives of this paper are the following: 1) map the spread of *S*. *alterniflora* in Yueqing Bay between 1993 and 2014, 2) analyze the rates and spatial patterns of *S*. *alterniflora* invasions, and 3) assess the relationship between *S*. *alterniflora* expansion, mangrove ecosystems and mudflat cultivation environment.

## Materials and Methods

### Ethics statement

No specific permissions were required for the field investigation in Yueqing Bay, China. We confirm that the field investigation in Yueqing Bay did not involve endangered or protected species.

### Study area

**Fig 1:**
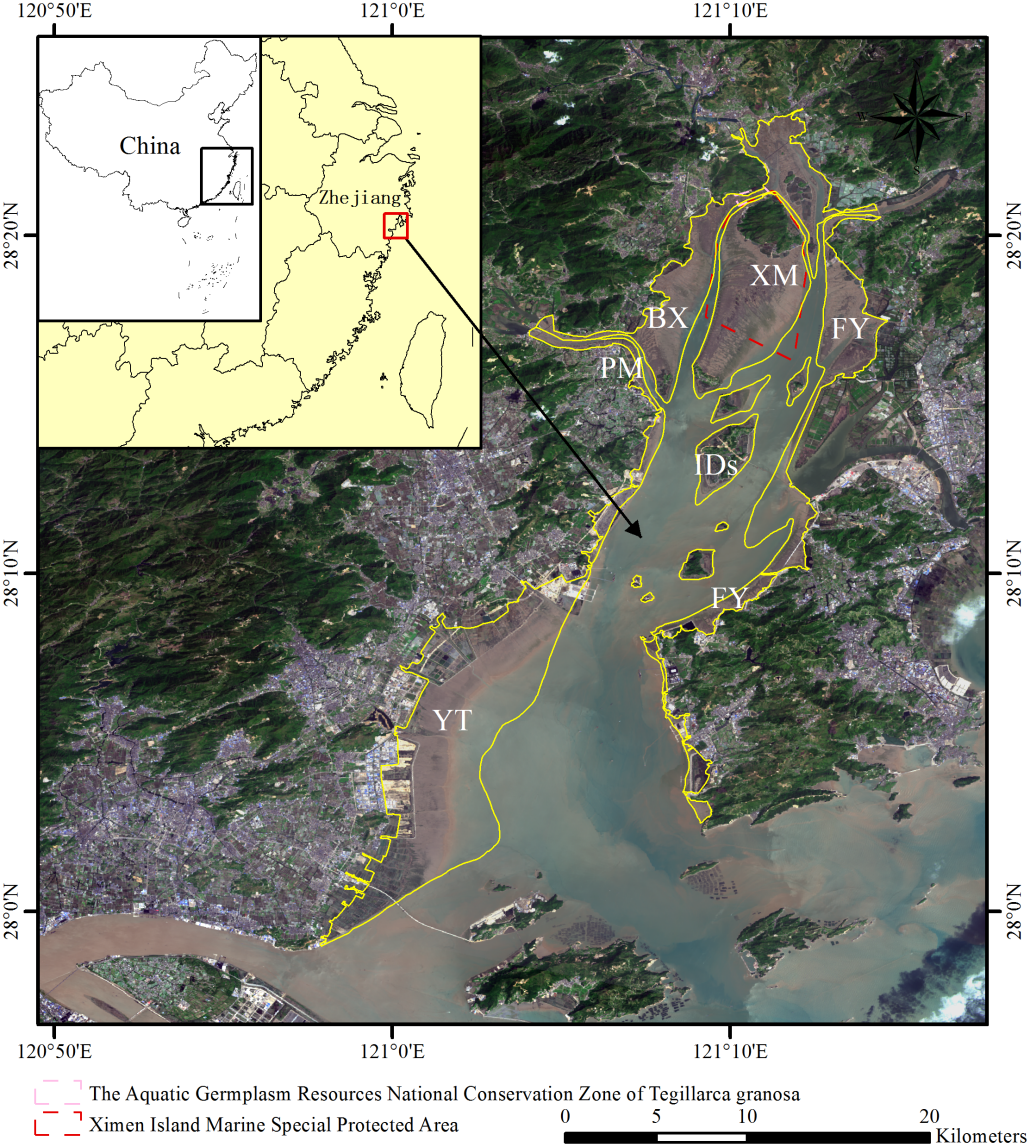
Study area in Yueqing, China. True color composite image of bands 3 (red), 2 (green), 1 (blue) from Landsat OLI imagery (2015). YT: Yantian, PM: Puma, BX: Baixi, XM: Ximen, FY: Fuyu, IDs: Islands. The Landsat images were downloaded from USGS.

The study area is located in southeast Zhejiang, China (27°59′09″- 28°24′16″N, 120°57′55″- 121°17′09″E) and has a northern subtropical oceanic climate, with an average annual rainfall of 1700 mm and a mean annual temperature of 17–18°C. The geomorphologic processes of Yueqing Bay are profoundly influenced by runoff and tidal (semidiurnal) currents. The average and maximum tidal ranges are 4.2 and 8.34 m, respectively. The study area covers 46360 ha. The 184.7-km-long coastline provides Yueqing Bay abundant marine resources and contains more than 10 islands (Fig 1). Historical mudflat cultivation was a crucial source of local income. In the northern part of Yueqing Bay, the Aquatic Germplasm Resources National Conservation Zone of *Tegillarca granosa* is an important mudflat cultivation base. The Ximen Island Marine Special Protected Area was the first national marine species protected area in Zhejiang province and contains the northernmost mangroves in China. After *S*. *alterniflora* was artificially introduced to Linkun Island in 1989 for coastal defense and tidal land reclamation, it was then introduced to Yueqing Bay. *S*. *alterniflora* was first found on the Yantian mudflat in 1993, and it expanded rapidly and has been threatening the local mangrove ecosystems and mudflat cultivation environment.

### Datasets

**Fig 2:**
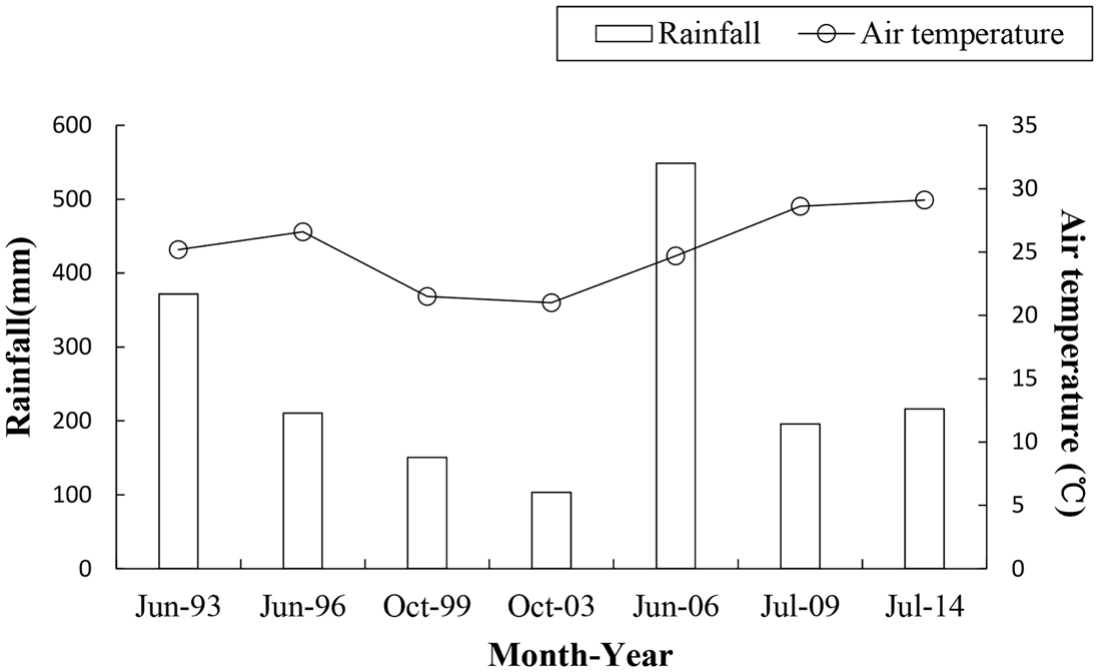
The rainfall and air temperature data in Yueqing Bay during the period from 1993 to 2014.

We used remote sensing data, including Landsat TM and SPOT 6 satellite images, to delineate the expansion of *S*. *alterniflora* (Table 1). All the Landsat images were freely downloaded from USGS (http://earthexplorer.usgs.gov/). The middle or peak of the growing season of *S*. *alterniflora* was summer to autumn. From 1993 to 2014, all images dated from summer to autumn after the onset of the rainy season when *S*. *alterniflora* was full developed (Fig 2). The images were acquired during low tides and were almost cloud-free. If few clouds were present, then they were masked. The Landsat TM and SPOT 6 images were geometrically corrected by a 1:50,000 scale nautical chart using the ENVI software. Quadratic polynomials were applied to the correction equations according to the distribution of the control points. All of the images were then resampled to a resolution of 30 m × 30 m, and the error was less than 0.5 pixels.

**Table 1:** Data Information.

Data	Region	Imaging date
Landsat TM (Multispectral: 30m)	118/40, 118/41	3-Jun-93
		27-Jun-96
		2-Oct-99
		21-Oct-03
		23-Jun-06
		17-Jul-09
SPOT 6 (Panchromatic: 1.5m, Multispectral: 6m)	Yueqing Bay	9-Jul-14
		9-Jul-14

Twelve landsat TM and two SPOT 6 images were used in our study.

### Data analysis

**Fig 3:**
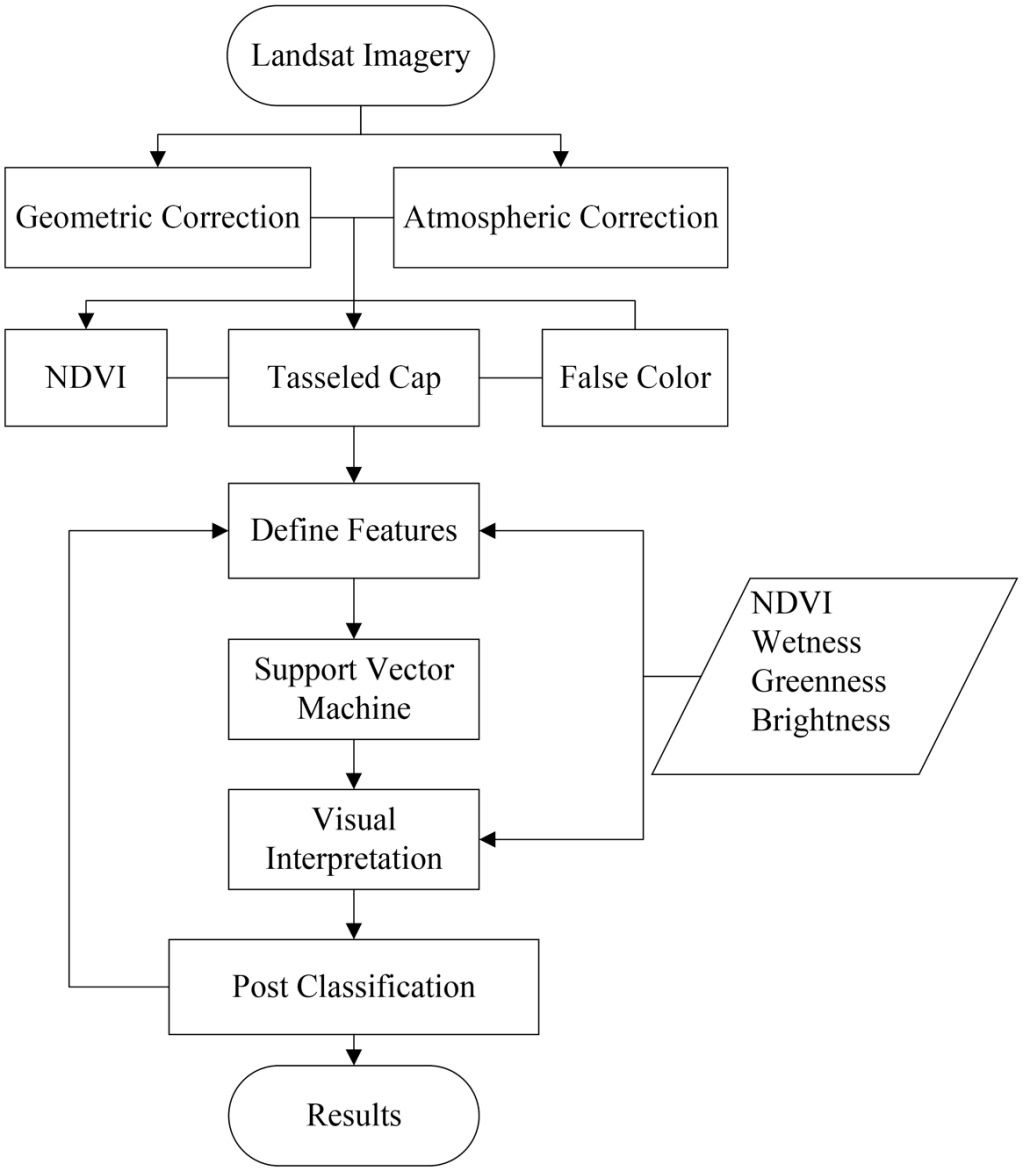
Flow chart showing the process of extracting *S*. *alterniflora* and other land cover classes using TM images from 1993–2009.

Multispectral Landsat TM data make generation of new sets of image components possible by spectral transformations [29]. To facilitate the interpretation of the twelve Landsat TM images, two spectral enhancement methods, the brightness, greenness, and wetness components of the Tasseled Cap (K-T) Transform and the Normal Difference Vegetation Index (NDVI) [30], were used on each image. Based on these image spectral enhancements, five classes, including water, *S*. *alterniflora*, mudflats, mudflat cultivation and all other cover types (‘other’, including urban, cropland, shrubs, and forest), were identified and selected as training samples. The training sites were used in an SVM classifier. We used the SVM to classify the multi-temporal stack of the twelve Landsat TM images with the ENVI software and to derive maps of the *S*. *alterniflora* expansion. The results of the classification were then integrated into ArcGIS to extract the vector of *S*. *alterniflora* and analyze the spatio-temporal dynamics of *S*. *alterniflora* (Fig 3).

OBIA was applied to the SPOT 6 satellite images. In OBIA, an image is segmented into groups of homogeneous pixels (image objects) that are classified according to color, shape, size, texture, pattern, context and other properties that contain both spectral and spatial information [24,27]. We used the eCognition 8.9 software for the multi-resolution segmentation of the SPOT 6 satellite images, which were then classified using a membership function nearest neighbor classification.

#### a. Image segmentation

**Fig 4:**
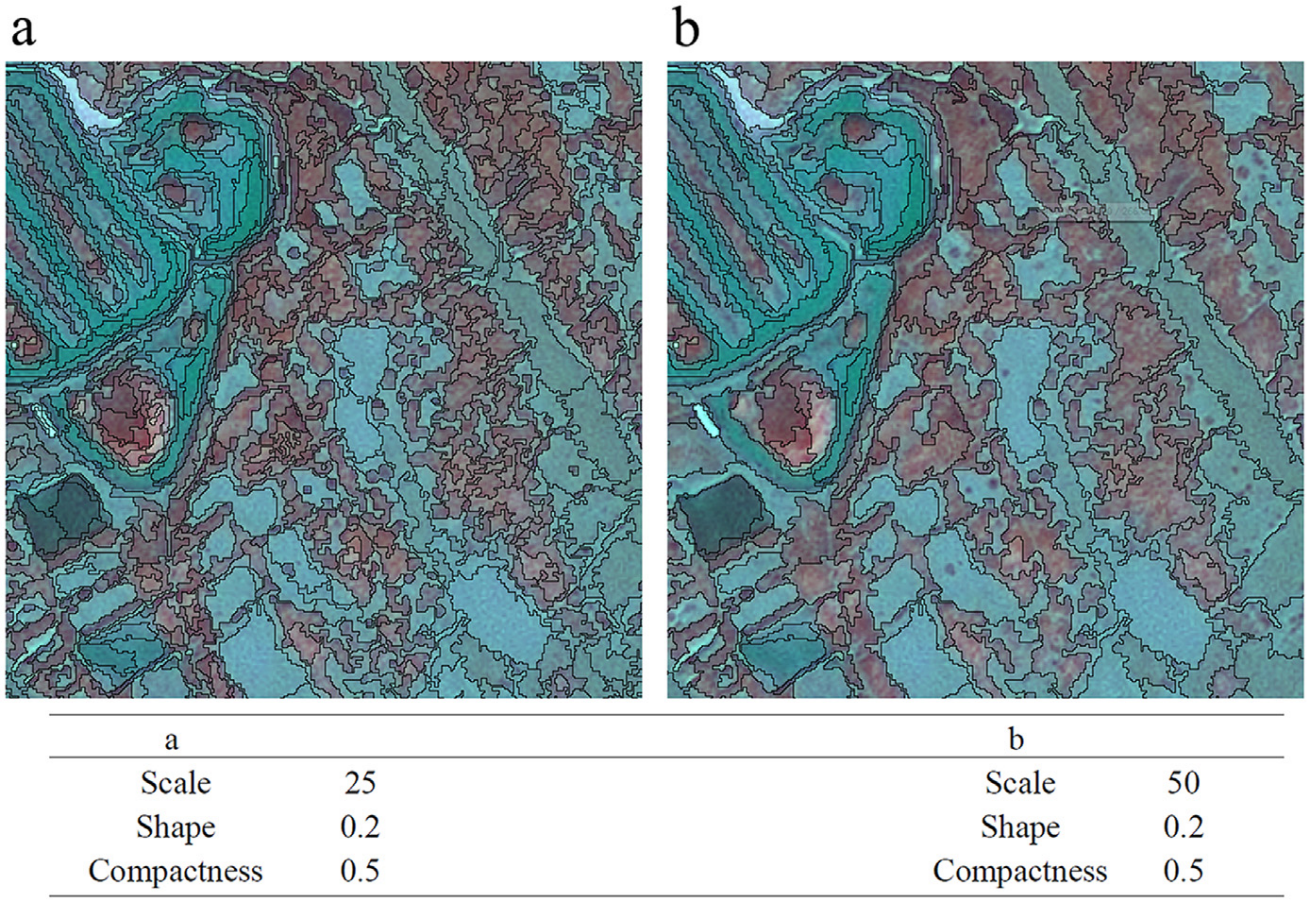
Examples of two segmentations at two scale levels. (a) Scale parameter 25; (b) Scale parameter 50, which is an example of above-segmentation. This two maps, the underlying RGB composite consists of a SPOT image, with composition R (4) G (3) B (2).

Multi-resolution segmentation is a leading function in image segmentation that separates an image into different regions or objects based on specific parameters [27,31,32]. We used this segmentation algorithm as implemented in Definiens Developer, which is based on the Fractal Net Evolution Approach (FNEA) [33]. The first step is to affirm three significant parameters: shape, compactness, and scale. The larger the scale parameter, the more objects can be merged, and the larger the objects grow [26]. The other two parameters, shape and compactness, determine how much the smoothness and compactness contribute to the shape heterogeneity. Although image segmentation is important to OBIA, there are no established criteria to determine the best parameters for segmentation [34]. Several groups of parameters were tested to determine the best scale by comparing segmented objects with uniform visual properties of the imagery (Fig 4). After testing different parameter values and evaluating them qualitatively, the level of segmentation was determined by a scale of 25, a shape factor of 0.2, and a compactness of 0.5.

#### b. Construction of the feature space

After the multi-resolution segmentation, we needed to define the feature space and the training samples (objects) that are used to extract the targets of interest. The feature space we used includes the spectrum (brightness, mean value of the bands), shape (length/width), the Digital Elevation Model (DEM), the Normalized Difference Vegetation Index (NDVI) and the Normalized Difference Water Index (NDWI).

#### c. Object-based classification methods

Two classification algorithms are used to assign classes to segmented objects in the eCognition software: the membership function and the nearest neighbor classifier. Rules and constraints can be defined in the membership function to control the classification procedure using the user's expert knowledge [27]. The classification using the nearest neighbor method is advantageous when using spectrally similar classes that are not well separated using only one or a few features. The two classifiers were applied to the segmented image objects using the feature space that was constructed in the last step.

**Fig 5:**
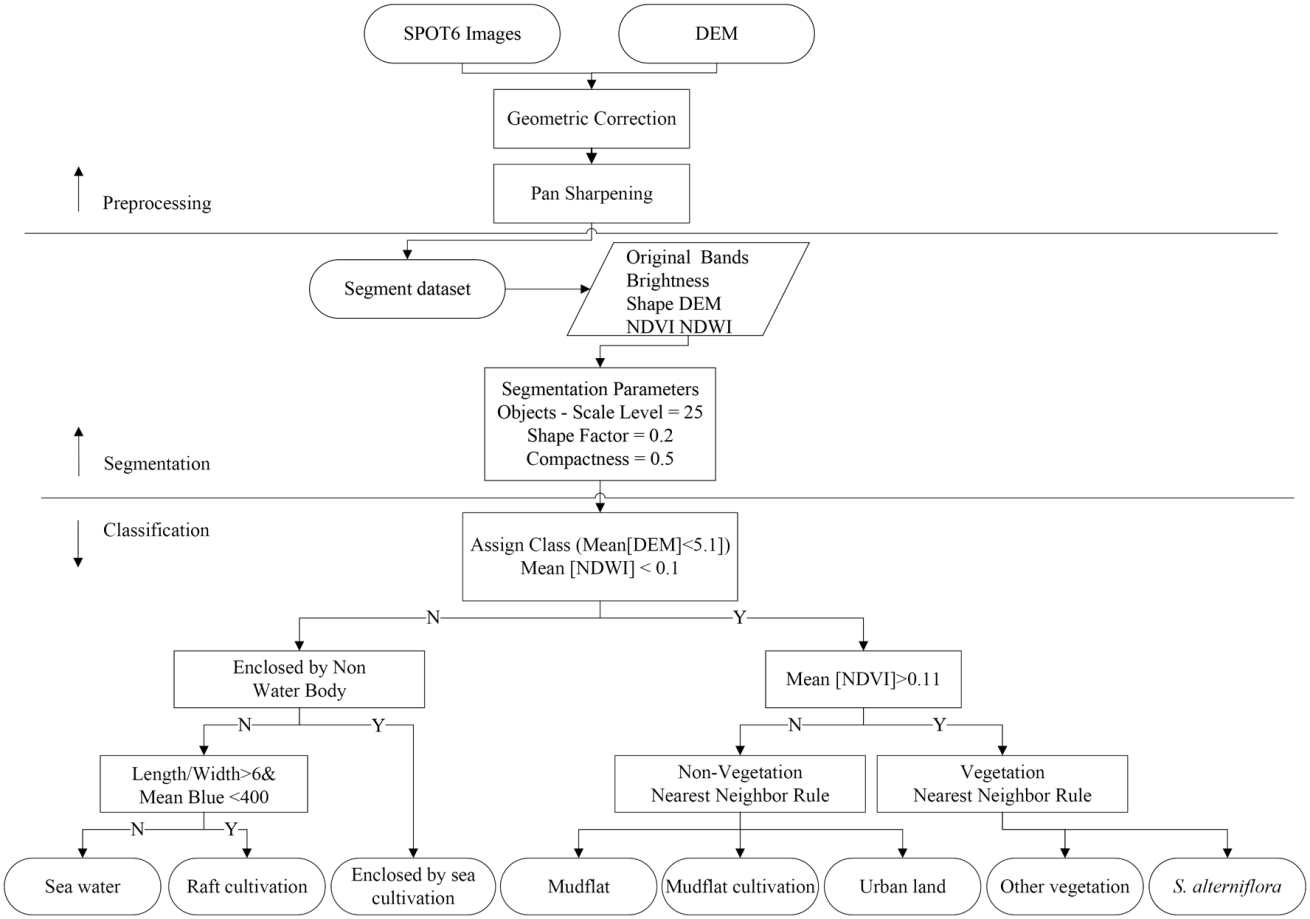
Flow chart showing the extraction of eight classes using a membership function and nearest neighbor classifiers with SPOT 6 imagery.

The mean change in elevation for all the image objects were less than 5.1 m. We then used an expert system rule that uses the mean of the NDWI values to extract the water bodies and non-water bodies. The water bodies that were enclosed by non-water bodies were classified as areas of enclosed sea cultivation. We found that mean shape index values (length/width) that are greater than 6 and mean blue band values that are less than 400 can effectively identify areas of raft cultivation from water bodies, the mean blue band values are digital numbers and they are not atmospherically corrected. We employed our expert knowledge in the membership function classifier to identify vegetation from non-water bodies. We determined that mean NDVI values greater than 0.11 can identify vegetation, and values less than 0.11 were non-vegetation. We then employed the nearest neighbor classifier to identify *S*. *alterniflora* and other vegetation (‘other’, i.e., cropland, mangrove, grasslands). The selected bands for the feature space include the mean of the red, green and blue bands, the mean of the brightness, the NDVI and the NDWI image. The objective of this component was to effectively discriminate *S*. *alterniflora* from other vegetation. The mudflat, mudflat cultivation and urban land areas were classified using the nearest neighbor method and the same feature space as the non-vegetation. Eight classes were identified in this model. After classifying them separately, we combined them using a GIS overlay function. Fig 5 shows a flow chart of the step-by-step procedure of these methods.

### In situ data and classification accuracy

Two data sets were used to validate our *S*. *alterniflora* map. First, an in situ field investigation was carried out in April and July 2014. A total of 64 sample plots were selected: 20 in Yantian, 8 in Puma, 16 in Baixi, 10 in Fuyu and 10 in Ximen. A Global Position System (GPS) was used to determine the coordinates of the sample points. Ten points were chosen on the edge of the *S*. *alterniflora* clumps in Ximen and surveyed with the GPS to acquire elevation data. Second, a UAV was used to collect very high resolution images (spatial resolution of 0.25 m) to improve the classification accuracy on the mudflat that was occupied by *S*. *alterniflora* and mudflat cultivation, which was difficult to access.

## Results

### Field verification and classification accuracy

We assessed the accuracy of initial classification from the high resolution images and the historical records, the results showed that the initial overall accuracy for the year 1993 was 81%, year 1996 was 78%, year 1999 was 83%, year 2003 was 74%, year 2006 was 84% and year 2009 was 82% for Landsat images (S1–S6 Tables). After initial classification, some misclassifications were corrected, and the overall accuracy for revised classification of different years reached to 80% to 90%. The in situ data and UAV images were used to verify the accuracy of the classification with a confusion matrix. Confusion matrices are used to provide an assessment of the correspondence between an image classification and ground data [35]. The overall classification accuracy for SPOT 6 was 87.4% (Table 2).

**Table 2:** Accuracy assessment.

Classified	Reference									Kappa
	MC	Sea	*S*. *alterniflora*	Mudflat	UL	OV	EBSC	RC	Total	index
MC	110	0	10	14	6	0	0	0	140	0.88
Sea	0	125	0	7	0	0	0	1	133	1
*S*. *alterniflora*	0	0	217	0	11	37	0	0	265	0.90
Mudflat	0	0	0	90	0	0	0	0	90	0.77
UL	10	0	7	0	54	3	0	0	74	0.71
OV	0	0	0	0	2	170	3	0	175	0.76
EBSC	2	0	0	2	0	0	22	0	26	0.87
RC	0	0	0	0	0	0	0	11	11	0.91
Total	122	125	234	113	73	210	25	12	0	

Confusion matrix, overall accuracy and kappa index produced by the OBIA classifier in eight classes (sea, raft cultivation, enclosed by sea cultivation, mudflat, mudflat cultivation, urban land, other vegetation. *S*. *alterniflora*) in 2014. Overall accuracy = 87.4%. Overall kappa statistics = 0.84. MC: Mudflat cultivation, UL: Urban land, OV: Other vegetation, EBSC: enclosed by sea cultivation. RC: Raft cultivation.

### Expansion of *S*. *alterniflora*

The interpretation of the imagery showed that there was less vegetation on the mudflats of Yueqing Bay before the introduction of *S*. *alterniflora*. *S*. *alterniflora* was artificially introduced in 1989 to Linkun Island (in the southern part of Yueqing Bay). Due to the small size of the clumps, the newly planted *S*. *alterniflora* could not be discerned on the mudflat of Yantian in the Landsat TM images until 1993. Between 1993 and 2014, *S*. *alterniflora*-dominated stands expanded in the study area from 4 ha to more than 2700 ha (Table 3). The expansion of *S*. *alterniflora* was relatively slow between 1993 and 1999, an increase of 269 ha with 44.8 ha per year, the invasion rate then increased rapidly between 1999 and 2009, an increase of 2163 ha with 216.3 ha per year. However, the rate of expansion began to slow down after 2009 (from 2009 to 2014), an increase of 270 ha with 54 ha per year (Table 3).

**Table 3:** Expansion of *S*. *alterniflora* in Yueqing Bay from 1993–2014.

Year	YT	PM	BX	XM	FY	IDs	Total	Spartina expansion(ha)	Percent growth of *S*. *alterniflora*
1993	4	0	0	0	0	0	4		
1996	49	0	0	0	24	23	96	92	2300
1999	96	12	4	2	114	41	269	173	180
2003	291	59	21	43	239	112	765	496	184
2006	525	74	39	206	244	117	1205	440	57
2009	1124	114	192	537	308	157	2432	1227	102
2014	835	100	363	685	571	148	2702	270	11

YT: Yantian, PM: Puma, BX: Baixi, XM: Ximen, FY: Fuyu, IDs: Islands.

**Fig 6:**
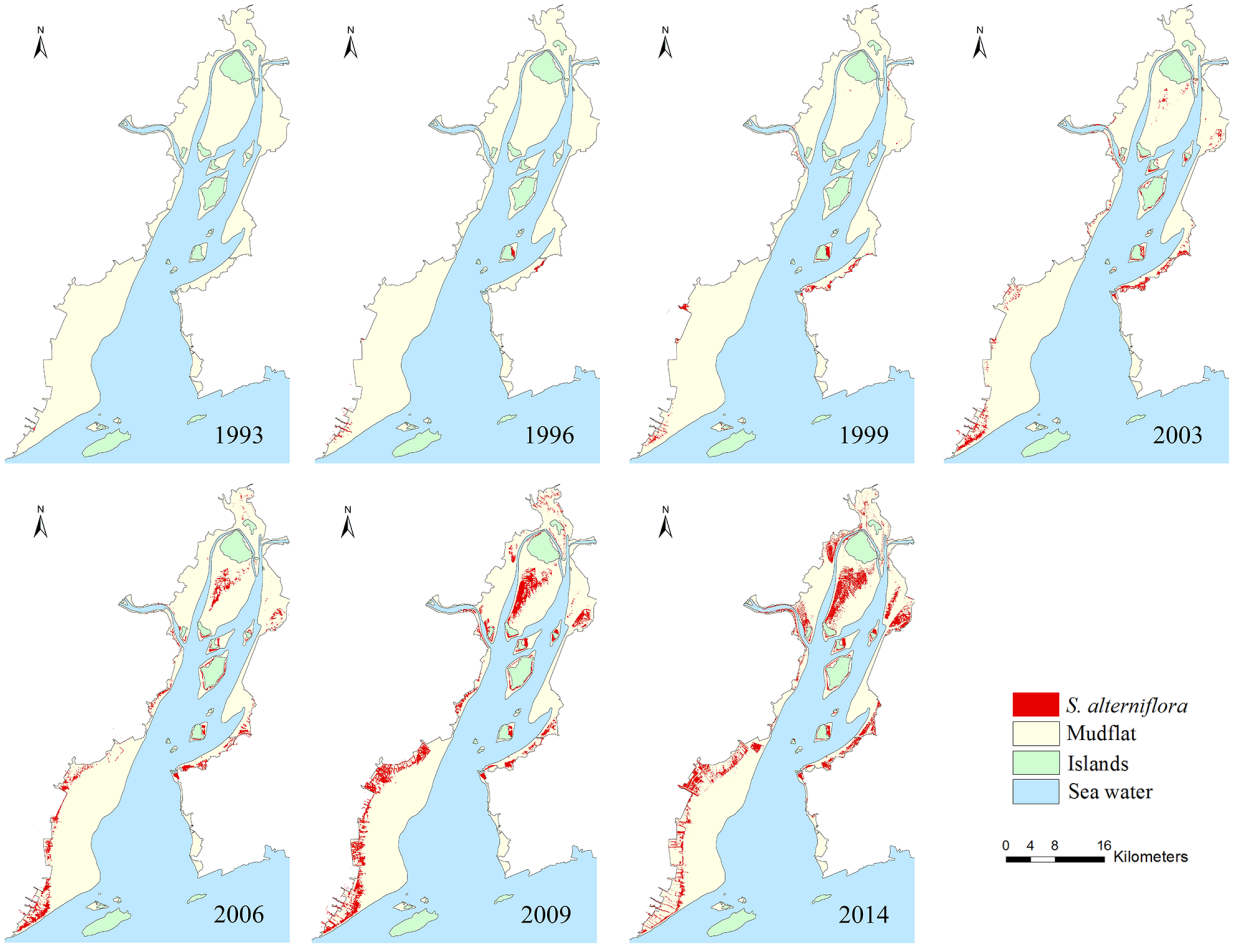
The rapid expansion of *S*. *alterniflora* from 1993 to 2014 in Yueqing Bay. Landsat TM images and SVM were used for the extraction of *S*. *alterniflora* between 1993 and 2009. The OBIA was applied to SPOT 6 images to map the distribution of *S*. *alterniflora* in 2014.

In 1993, most of the *S*. *alterniflora*-dominated areas were located in a few small stands on the Yantian mudflat. *S*. *alterniflora* expanded between 1993 and 1996, particularly on the Yantian and Fuyu mudflats and the islands. Between 1996 and 1999, a few new *S*. *alterniflora* areas appeared on the Puma, Baixi and Ximen mudflats (Fig 6).

The rapid expansion of *S*. *alterniflora* after 1999 had two well-defined patterns. Between 1999 and 2003, there was a large expansion of *S*. *alterniflora* in the areas of Yantian, Puma and Fuyu. Another large increase in the area of *S*. *alterniflora* occurred on the Ximen and Baixi mudflats. Between 2003 and 2014, the expansion of *S*. *alterniflora* mainly occurred on the Ximen mudflat (Fig 6).

**Fig 7:**
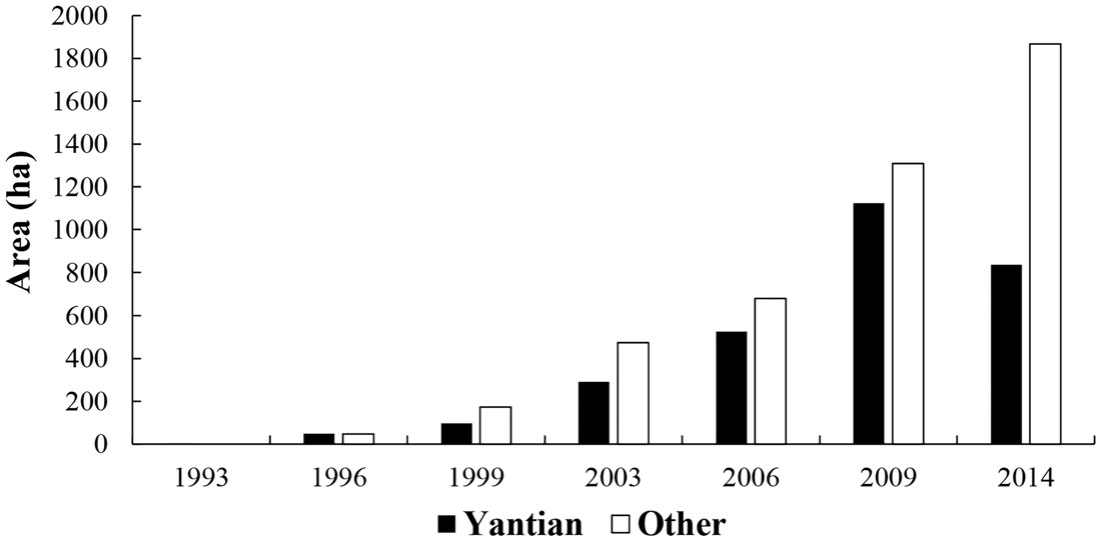
Areas (ha) of *S*. *alterniflora* in Yantian and other regions from 1993 to 2014.

The rate of expansion of *S*. *alterniflora* began to slow down from 2009 to 2014. The precent growth of *S*. *alterniflora* in Yueqing Bay was only 15%. In particular, the area of the *S*. *alterniflora* communities on the Yantian and Puma mudflats decreased by 275 ha and 14 ha, respectively (Figs 6 and 7).

### Current status of *S*. *alterniflora*, mangrove and mudflat cultivation areas

**Fig 8:**
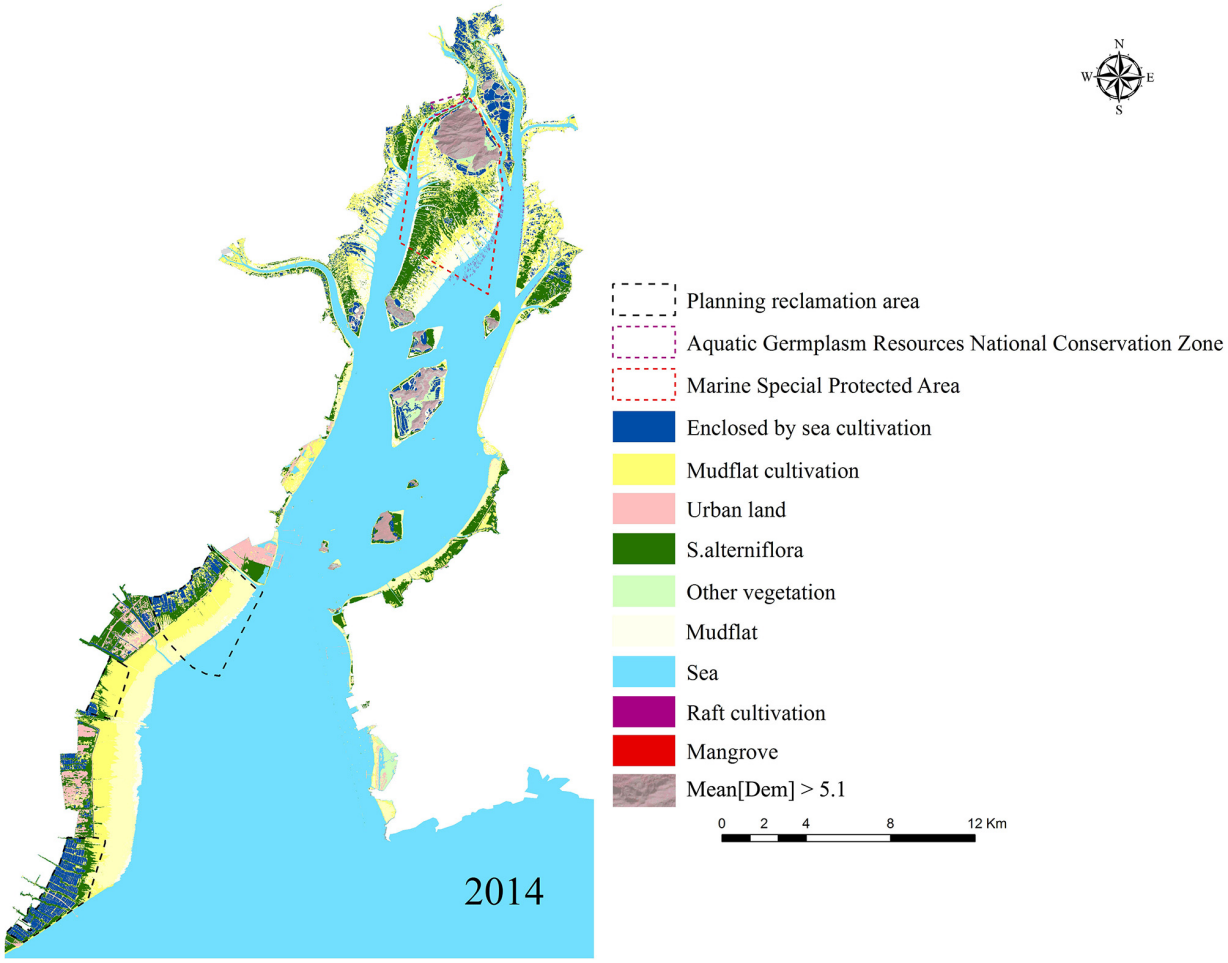
Final classification map based on the flow chart (OBIA). The SPOT 6 images were downloaded from Astrium GEO-information Services.

In 2014, *S*. *alterniflora* occupied 2702 ha of the mudflats of Yueqing Bay. Mangroves covered an area of 2.76 ha on the mudflat of the Ximen Island Marine Special Protected Area. The total cultivated area was 6546.1 ha, which includes 4766.71 ha of mudflat cultivation and 1779.39 ha of enclosed sea cultivation (Fig 8).

In Ximen and Baixi, more than 40% of the *S*. *alterniflora* communities were located on the mudflat in 2014. Over the past fifteen years (1999–2014), the area of *S*. *alterniflora* increased by 69.47 ha per year; the mean annual expansion rate was 41%, which is much faster than in the other areas of Yueqing Bay. Two national conservation zones are located in Ximen and Baixi, and *S*. *alterniflora* covered 531.26 ha and 8.7 ha of these two zones, which represent 17.2% and 12.7% of their areas, respectively. In Puma, the area of *S*. *alterniflora* expanded southeast along the river from 12 ha in 1999 to 100 ha in 2014, with a mean annual expansion rate of 15%. In Yantian, *S*. *alterniflora* was located on the mudflat and covered a total area of 835 ha; the mean annual expansion rate was 29%. In Fuyu, *S*. *alterniflora* was located on the mudflat and covered a total area of 571 ha, and the mean annual expansion rate was 19%.

## Discussion

The results indicated that the expansion of *S*. *alterniflora* occurred in three distinct stages. The first stage was the colonization. In 1993, *S*. *alterniflora* was only present in a small area (4 ha) in Yantian. Between 1996 and 1999, *S*. *alterniflora* began to appear in other areas. One common feature of invasions is the lag time between colonization and rapid expansion [36]. For example, the lag time of the *S*. *alterniflora* expansion in Willapa Bay, Washington was nearly 50 years [37]. In the Jiuduansha shoals, a lag was also observed in 1998–2000 [20]. In Yueqing Bay, the area of *S*. *alterniflora* increased from 4 ha in 1993 to 96 ha in 1996, so the lag time in the study area could be defined in this period, the lag time can be interpreted mainly as the evolution of adaptations to the new habitat, and an Allee effect has slowed the rate of spread of the invasion in the early stage [38].

The second stage was the rapid range expansion (1999–2009). After 1999, the expansion rate of *S*. *alterniflora* in Yueqing Bay increased and reached a maximum in the period from 2006 to 2009. The rapid expansion of *S*. *alterniflora* requires both sexual and asexual reproduction [39], and *S*. *alterniflora* may spread by continuous long distance dispersal as well as by short-distance dispersal with lateral expansion of the established population [40]. Our results indicated that asexual reproduction was the main source of recruitment and that short-distance dispersal with lateral expansion of the established population was the main initial spreading pattern for *S*. *alterniflora*. Sexual reproduction also played an important role in connecting isolated populations through seed dispersal and in colonizing new habitats and expanding the range in the study area.

**Fig 9:**
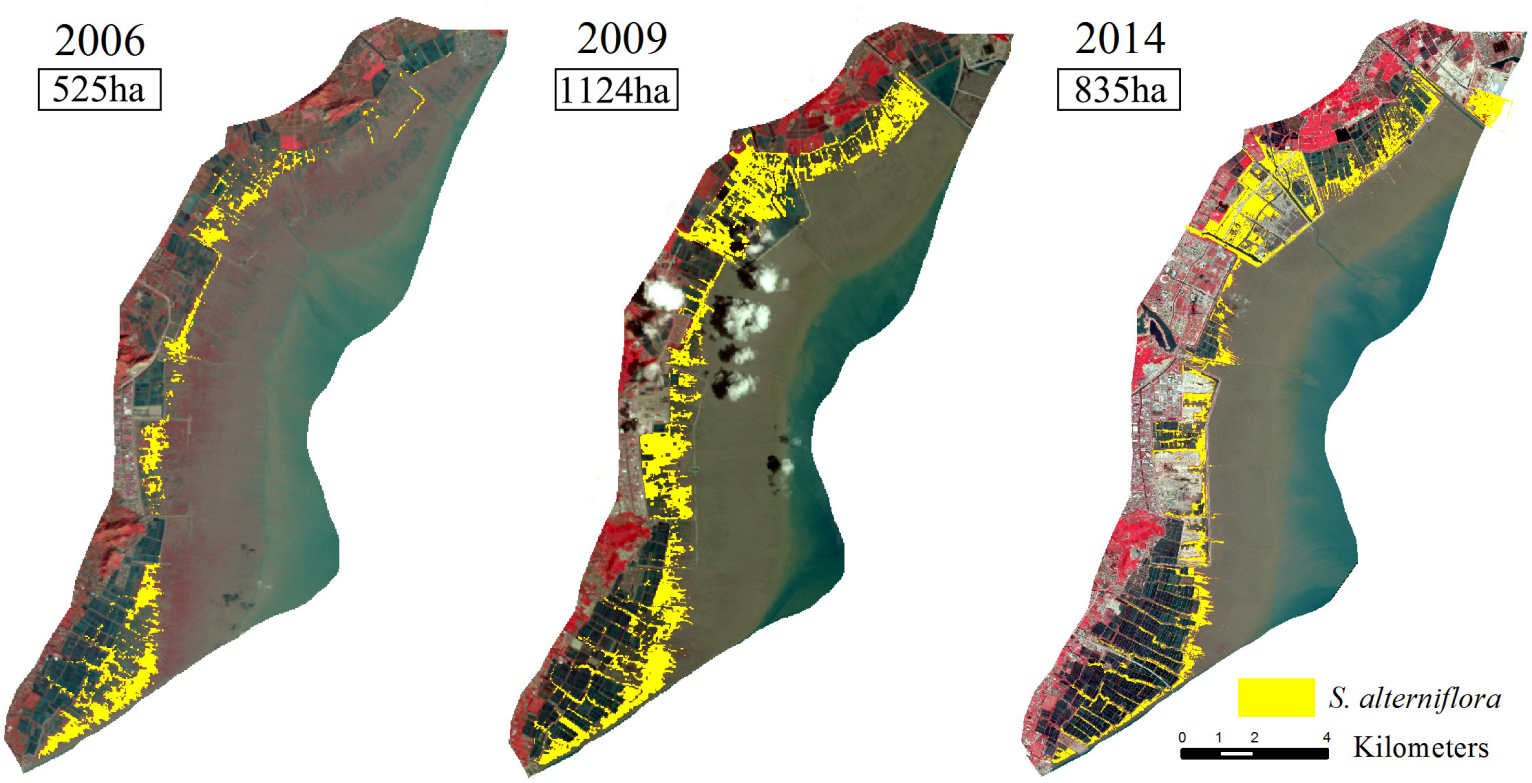
The expansion of *S*. *alterniflora* in Yantian from 2006 to 2014. Color composite images (bands 4, 3, 2) from TM (2006 and 2009) and SPOT 6 (2014) with the results of classification. The landsat TM images were downloaded from USGS. The SPOT 6 images were downloaded from Astrium GEO-information Services.

The third stage, after 2009, included the lowest rate of expansion. The rate of *S*. *alterniflora* expansion began to slow down in Yueqing Bay, especially on the Yantian mudflat, where the area of *S*. *alterniflora* decreased by 289 ha. This decrease and the low expansion rate of *S*. *alterniflora* were most likely caused by two processes. First, changes in the landscape pattern due to human activities which affected plant invasions [41]. The sea dike and aquaculture areas can acted as sources of propagules that invaded adjacent areas, and large numbers of *S*. *alterniflora* are commonly located around these areas. In addition, human activities in Yantian resulted in increasing tidal land reclamation and aquaculture in areas of the mudflat that were previously occupied by *S*. *alterniflora* (Fig 9). Second, several studies have shown that the expansion rate of *S*. *alterniflora* would begin slowing when the coastal topographical ecological niches had been occupied by this exotic plant. In Jiangsu, after most of the suitable areas on the mudflat had been occupied by *S*. *alterniflora*, the expansion rate of *S*. *alterniflora* was only 10%. Research has indicated that *S*. *alterniflora* occupied areas with elevations of 2–3 m [12]. Our results from Yueqing Bay indicated that there was less lateral expansion after the ecological niches were occupied by *S*. *alterniflora* and that the lowest elevation of *S*. *alterniflora* in the salt marsh was 1.4 m above mean sea level.

The original purpose of introducing *S*. *alterniflora* to Yueqing Bay was for seashore stabilization, tidal land reclamation, and defense against typhoons [7,9,13]. Several studies have indicated that using *S*. *alterniflora* plantations for ecological engineering can provide both ecological and economic benefits [7,13,17,42,43]. For example, in 1994, a 4000-m-long dike in Yueqing Bay was seriously damaged by a large typhoon. In contrast, 600 m of an 800-m-long dike in Wenling, near Yueqing Bay, that was covered by dense growth of *S*. *alterniflora* remained in good condition. The planting of *S*. *alterniflora* could save more than 6,444,000 USD over the cost of building a dike [9]. However, several recent studies have reported that *S*. *alterniflora* negatively affects ecosystem functioning and biodiversity [6,13,14,16].

**Fig 10:**
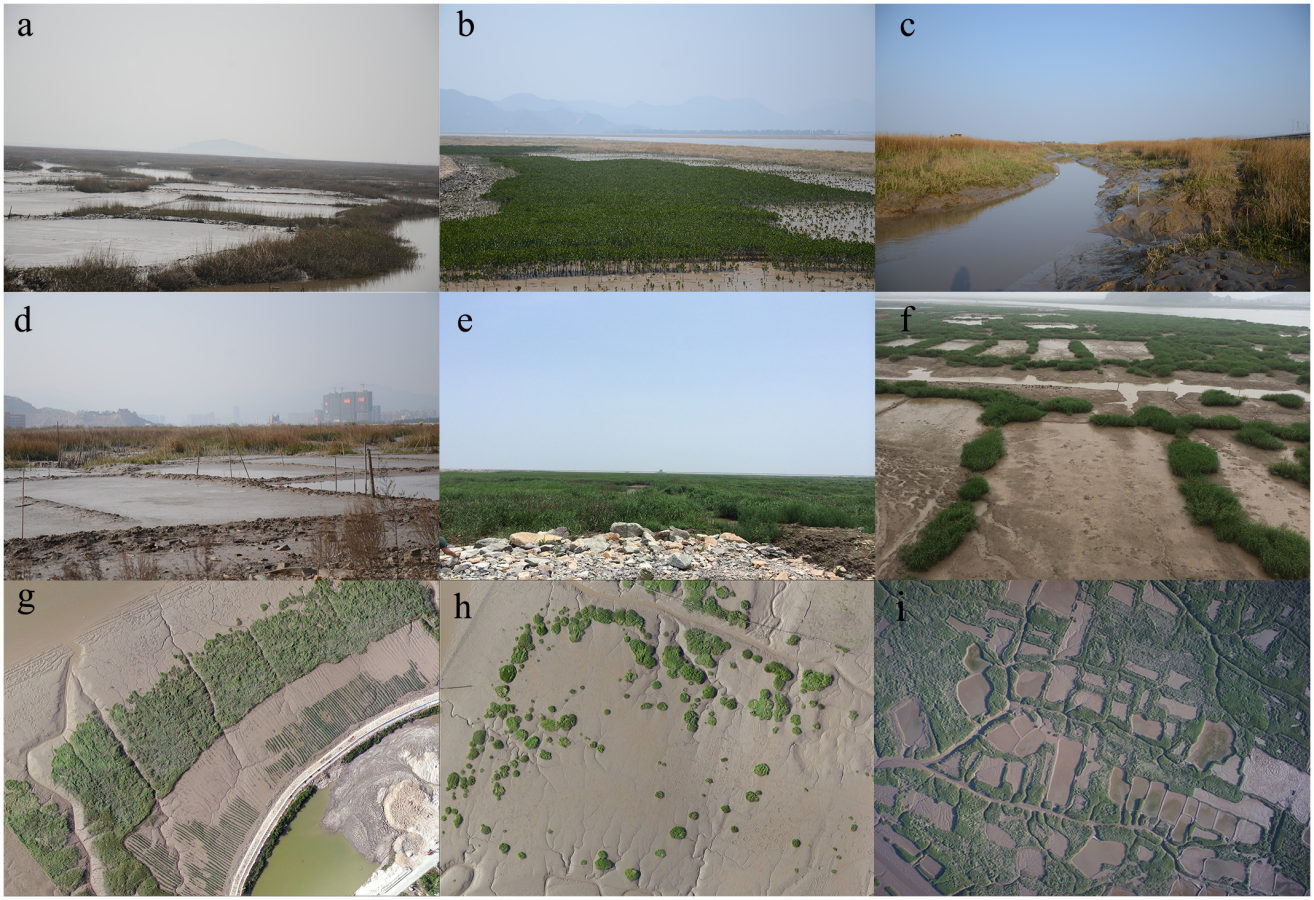
The invasion of *S*. *alterniflora* in Yueqing Bay with in situ photos (a-f) and the UAV images (g-i). (a) *S*. *alterniflora* located around the mudflat area in Ximen; (b) *S*. *alterniflora* covered almost all of the bare mudflats around the mangrove seedlings area; (c) Tidal creeks in *S*. *alterniflora* salt-marshes; (d) and (e) The invasion of *S*. *alterniflora* were in the reclamation area near the urban land; (f) The small *S*. *alterniflora* patches; (g) The invasion of *S*. *alterniflora* covered the mudflats around the mangrove seedlings area with UAV images in the same region of (b); (h) The small *S*. *alterniflora* patches can be identified in the UAV images, which is a very important data in further study to model population and community dynamics and to study the interactions between spatial pattern and ecosystem processes of *S*. *alterniflora* in Yueqing Bay; (i) *S*. *alterniflora* covered the mudflats around the mudflat cultivation area imaged by UAV platform.

Our research in Yueqing Bay indicated that the invasion of *S*. *alterniflora* may threaten mangrove ecosystems and coastal aquaculture. High spatial resolution imagery and OBIA have been successfully used to detect monospecific vegetation stands in several ecosystems, including coastal areas [24]. In our research, SPOT 6 imagery and OBIA were used to map the distribution of *S*. *alterniflora* in Yueqing Bay. The results of the investigation showed that monospecific stands of *S*. *alterniflora* covered almost all of the bare mudflats around the mangrove forests (Fig 10). Previous research has demonstrated that *S*. *alterniflora* could gradually replace mangroves in mid-salinity mudflats without appropriate intervention, especially in areas where human disturbance has created gaps in the mangrove canopy [14]. The mangrove in Ximen Island was successfully transplanted during the 1950s from Fujian Province [44]. The invasion of *S*. *alterniflora* in the Ximen Marine Special Protected Area, which contains mangrove ecosystems, may threaten the long-term success of these restoration efforts.

Mudflat cultivation has a long history in Yueqing Bay, but the invasion of *S*. *alterniflora* has occupied much of the mudflat area (Fig 10). The Yueqing Bay Aquatic Germplasm Resources National Conservation Zone of *Tegillarca granosa*, which is one of the most significant mudflat cultivation bases, had been negatively affected by the invasion of *S*. *alterniflora*. The *S*. *alterniflora* has occupied much of the area of mudflat cultivation both on and below the sediment surface because of its rapid expansion (Fig 10), and the spread of *S*. *alterniflora* is likely to produce massive changes from mudflat to vegetated marsh systems with accompanying declines in animal biomass and density, which negatively influences the trophic and habitat support for mudflat cultivation [16]. In Yueqing Bay, The observed transformations in the distribution of the *S*. *alterniflora* may cause trophic level changes and habitat losses of mudflat cultivation.

Ecological engineering with *S*. *alterniflora* transplantations can cause different ecological and economic effects in different areas [18]. The conservation plans for Yueqing Bay should consider both the positive and negative effects of *S*. *alterniflora*, and the management policy should consider the different regional characteristics. For example, in Yantian, the expansion rate of *S*. *alterniflora* has slowed, and *S*. *alterniflora* contributed to protection against typhoons and the reclamation of tidal land (Fig 10). Hence, the management policies for this area should maintain the expansion of *S*. *alterniflora* in the reclaimed areas. However, in the Ximen Marine Special Protected Area, *S*. *alterniflora* continue to expand rapidly, which threatens the mangrove ecosystems and mudflat cultivation. Several studies have indicated that physical, chemical and biological control measures can be used for control invasive *S*. *alterniflora* [45,46]. These policies should be directed at eliminating *S*. *alterniflora* from Ximen Island.

The continued monitoring of the *S*. *alterniflora* expansion is important. Landsat TM images provide a rich historical data source, and SVM was a promising technique to map the invasion of *S*. *alterniflora*. Landsat TM images had limited usefulness in mapping *S*. *alterniflora* in mixed stands of *S*. *alterniflora* and native forests, which could not be reliably detected because of the spatial and spectral resolutions of Landsat's TM sensors. To delineate the distribution of *S*. *alterniflora*, we used UAV and SPOT 6 images. It clearly showed that the higher spatial resolution images (UAV images) can provide more useful information for the classification [47]; even very small *S*. *alterniflora* patches (0-1m^2^) can be identified in the UAV images (Fig 10). By the detection through the UAV images, the small patches can be eradicated in the early stage of expansion, which is important for conservation, and the information of small patches can be used to model population and community of *S*. *alterniflora* dynamics, and future research should also focus on quantifying the proportions of sexual and asexual reproduction with the measured data. OBIA was applied to the high spatial resolution images, which decreased the “salt and pepper” phenomenon compared to pixel-based classification [24]. In our study, computer memory limited the segmentation results because of the large numbers of objects that needed to be processed. Similar issues have been reported in other studies [27]. To improve the accuracy, we will explore and test additional classifications after performing the image segmentation in future research. The images and analysis methods we used in this study could provide an effective toolset to analyze the spatial distribution and expansion of *S*. *alterniflora* at regional scales.

## Supporting Information

S1 File The granted permission of Landsat images. (PDF)

S2 File The granted permission of SPOT 6 images. (PDF)

S1 Table Accuracy assessment for the classification of Landsat images in 1993. (DOCX)

S2 Table Accuracy assessment for the classification of Landsat images in 1996. (DOCX)

S3 Table Accuracy assessment for the classification of Landsat images in 1999. (DOCX)

S4 Table Accuracy assessment for the classification of Landsat images in 2003. (DOCX)

S5 Table Accuracy assessment for the classification of Landsat images in 2006. (DOCX)

S6 Table Accuracy assessment for the classification of Landsat images in 2009. (DOCX)
