# Supplementary material for: Monitoring the Invasion of Spartina alterniflora from 1993 to 2014 with Landsat TM and SPOT 6 Satellite Data in Yueqing Bay, China
Source: PLoS One. 2015 Aug 11;10(8):e0135538. doi: 10.1371/journal.pone.0135538 (PMC4532505; doi:10.1371/journal.pone.0135538)
Supplement: S1 File — (PDF) [file pone.0135538.s001.pdf]

Data Citation

There are no restrictions on the use of data received from the U.S. Geological Survey's Earth Resources Observation and Science (EROS) Center or NASA's Land Processes Distributed Active Archive Center (LP DAAC), unless expressly identified prior to or at the time of receipt. Depending on the product source, we request that the following statements be used when citing, copying, or reprinting data:

USGS Products

Acknowledgement:

Data available from the U.S. Geological Survey.

See USGS Visual Identity System Guidance for further details.

Questions concerning the use or redistribution of USGS data should be directed to: ask@usgs.gov or 1-888-ASK-USGS (1-888-275-8747).
