# Supplementary material for: Monitoring the Invasion of Spartina alterniflora from 1993 to 2014 with Landsat TM and SPOT 6 Satellite Data in Yueqing Bay, China
Source: PLoS One. 2015 Aug 11;10(8):e0135538. doi: 10.1371/journal.pone.0135538 (PMC4532505; doi:10.1371/journal.pone.0135538)

Medium Directory

Position	*Item*	*Description*
1	1	PROD_SPOT6_001<br>VOL_SPOT6_001_A<br>IMG_SPOT6_P_001_A : SPOT 6 2014-07-09:02:06:13.9 SENSOR P<br>IMG_SPOT6_MS_001_A : SPOT 6 2014-07-09:02:06:13.9 SENSOR MS

Number of product(s) : 1

Product PROD_SPOT6_001/VOL_SPOT6_001_A/IMG_SPOT6_P_001_A

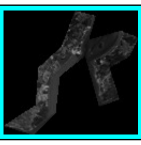	**DIM_SPOT6_P_201407090206139_SEN_14706275-0_01.XML**

Dataset Id	**SEN_SPOT6_20140709_020616900_000**
product code	**undefined**
Unitary Production Date	**2014-07-22T11:03:08.00Z**
Image dimensions	**19469 rows x 20973 columns**
Acquisition date	**2014-07-09 02:06:13.9**
Platform name	**SPOT 6**
Spectral Mode	**PMS**
Spectral Processing	**P**
Processing level	**SENSOR**
Number of spectral bands	**1**
Spectral bands id	**P**
Solar irradiance value of raw radiometric Band	**1706.514896**
Orientation angle	**+114.43°**
Incidence angle	**+19.67°**
Sun Azimuth	**+95.62°**
Sun Elevation	**+63.58°**

Image Centre Location

Row	Column	Latitude	Longitude
**9735**	**10487**	**N028°06'39"**	**E121°04'18"**

Image Vertex Location

Row	Column	Latitude	Longitude
**1**	**1**	**N028°15'40"**	**E120°53'19"**
**1**	**20973**	**N028°14'52"**	**E121°15'05"**
**19469**	**20973**	**N027°57'40"**	**E121°15'00"**
**19469**	**1**	**N027°58'25"**	**E120°53'25"**

Files provided within the product :

Name
**LINEAGE/HEIGHT_GLOBE_DIM.XML**
**LINEAGE/HEIGHT_SRTM_DT1_DIM.XML**
**RPC_SPOT6_P_201407090206139_SEN_14706275-0_01.XML**
**LINEAGE/PROCESSING_SPOT6_P_201407090206139_SEN_14706275-0_01_DIM.XML**
**LINEAGE/STRIP_DS_SPOT6_201407090206139_YZ1_YZ1_YZ1_YZ1_E121N28_01790_DIM.XML**
**RPC_SPOT6_P_201407090206139_SEN_14706275-0_01.XML**
**LINEAGE/PROCESSING_SPOT6_P_201407090206139_SEN_14706275-0_01_DIM.XML**
**LINEAGE/STRIP_DS_SPOT6_201407090206139_YZ1_YZ1_YZ1_YZ1_E121N28_01790_DIM.XML**
**LINEAGE/HEIGHT_GLOBE_DIM.XML**
**LINEAGE/HEIGHT_SRTM_DT1_DIM.XML**

Histograms PROD_SPOT6_001/VOL_SPOT6_001_A/IMG_SPOT6_P_001_A

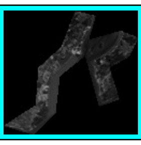	**DIM_SPOT6_P_201407090206139_SEN_14706275-0_01.XML**

Histogram for Band P :

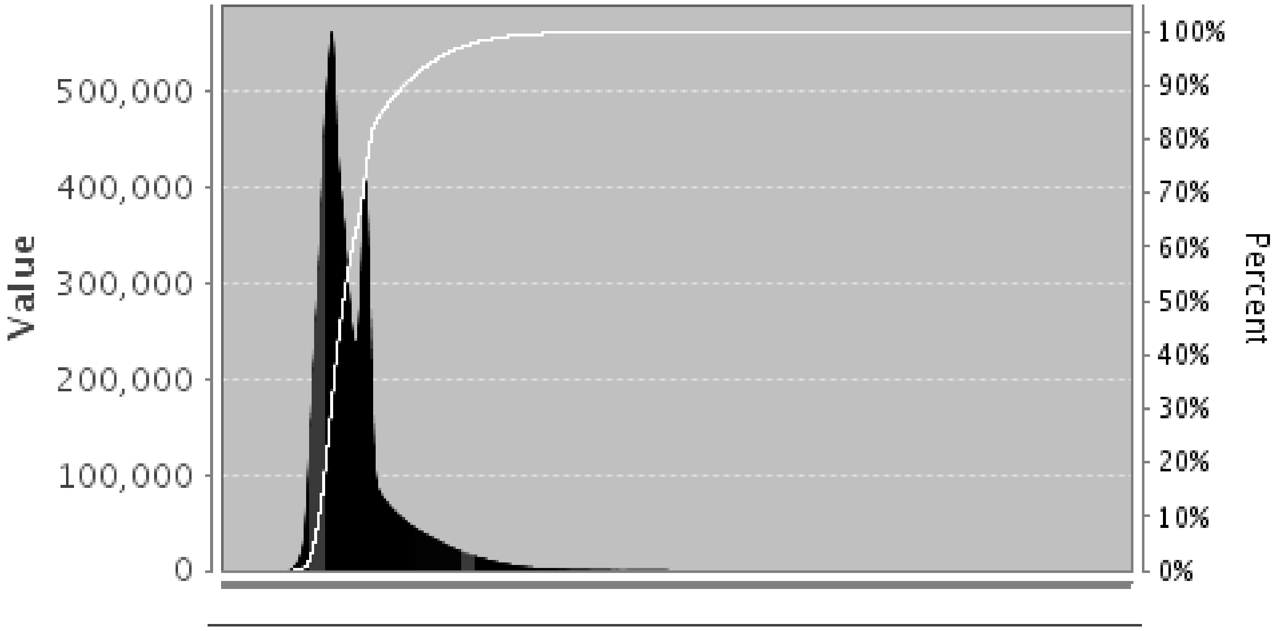

Product PROD_SPOT6_001/VOL_SPOT6_001_A/IMG_SPOT6_MS_001_A

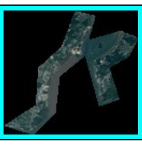	**DIM_SPOT6_MS_201407090206139_SEN_14706275-0_01.XML**

Dataset Id	**SEN_SPOT6_20140709_020616900_000**
product code	**undefined**
Unitary Production Date	**2014-07-22T11:03:08.00Z**
Image dimensions	**4867 rows x 5243 columns**
Acquisition date	**2014-07-09 02:06:13.9**
Platform name	**SPOT 6**
Spectral Mode	**PMS**
Spectral Processing	**MS**
Processing level	**SENSOR**
Number of spectral bands	**4**
Spectral bands id	**B2 B1 B0 B3**
Solar irradiance value of raw radiometric Band	**1540.494123 1826.087443 1982.671954 1094.747446**
Orientation angle	**+114.43°**
Incidence angle	**+19.67°**
Sun Azimuth	**+95.62°**
Sun Elevation	**+63.58°**

Image Centre Location

Row	Column	Latitude	Longitude
**2434**	**2622**	**N028°06'39"**	**E121°04'18"**

Image Vertex Location

Row	Column	Latitude	Longitude
**1**	**1**	**N028°15'39"**	**E120°53'20"**
**1**	**5243**	**N028°14'52"**	**E121°15'05"**
**4867**	**5243**	**N027°57'40"**	**E121°15'00"**
**4867**	**1**	**N027°58'25"**	**E120°53'26"**

Files provided within the product :

Name
**LINEAGE/HEIGHT_GLOBE_DIM.XML**
**LINEAGE/HEIGHT_SRTM_DT1_DIM.XML**
**RPC_SPOT6_MS_201407090206139_SEN_14706275-0_01.XML**
**LINEAGE/PROCESSING_SPOT6_MS_201407090206139_SEN_14706275-0_01_DIM.XML**
**LINEAGE/STRIP_DS_SPOT6_201407090206139_YZ1_YZ1_YZ1_YZ1_E121N28_01790_DIM.XML**
**RPC_SPOT6_MS_201407090206139_SEN_14706275-0_01.XML**
**LINEAGE/PROCESSING_SPOT6_MS_201407090206139_SEN_14706275-0_01_DIM.XML**
**LINEAGE/STRIP_DS_SPOT6_201407090206139_YZ1_YZ1_YZ1_YZ1_E121N28_01790_DIM.XML**
**LINEAGE/HEIGHT_GLOBE_DIM.XML**
**LINEAGE/HEIGHT_SRTM_DT1_DIM.XML**

AstroTerra Image Facilities
IMF

Histograms PROD_SPOT6_001/VOL_SPOT6_001_A/IMG_SPOT6_MS_001_A

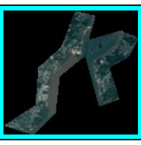	**DIM_SPOT6_MS_201407090206139_SEN_14706275-0_01.XML**

Histogram for Band B2 :

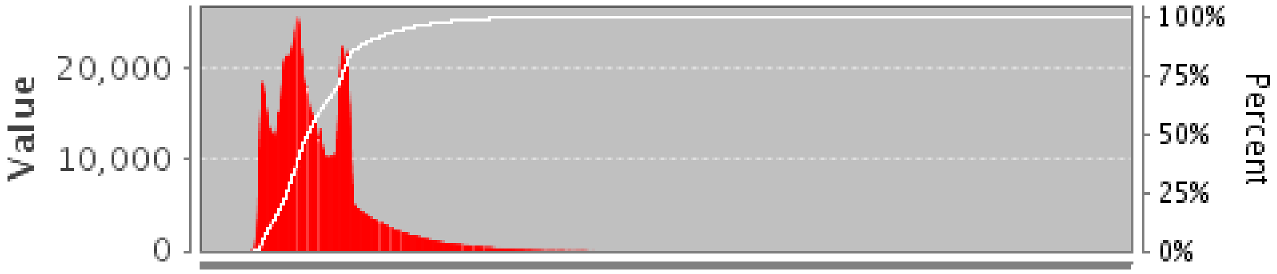

Histogram for Band B1 :

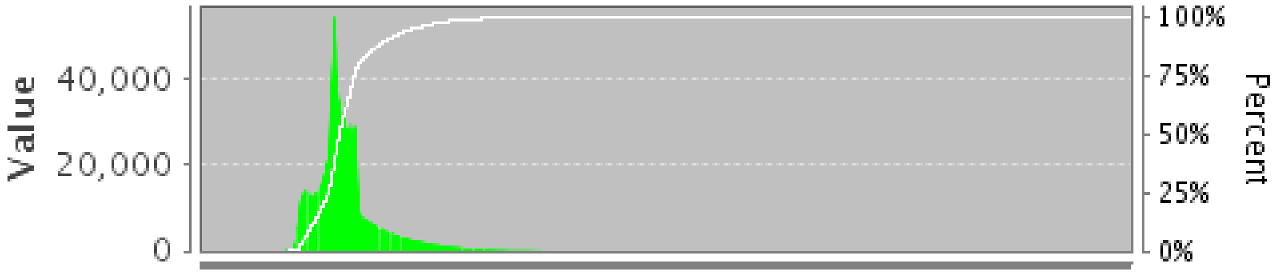

Histogram for Band B0 :

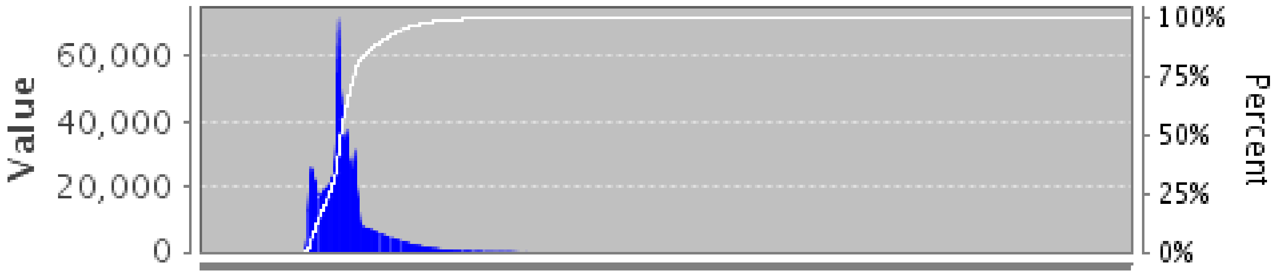

Histogram for Band B3 :

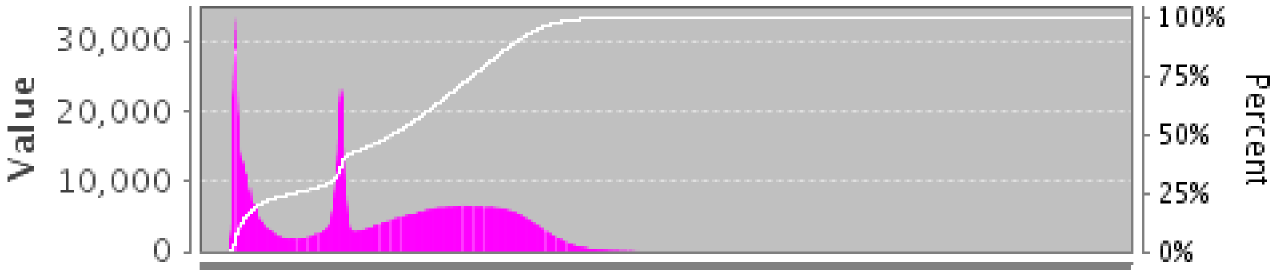

Supplement: S2 File — (PDF) [file pone.0135538.s002.PDF]
