# Supplementary material for: Monitoring the Invasion of Spartina alterniflora from 1993 to 2014 with Landsat TM and SPOT 6 Satellite Data in Yueqing Bay, China
Source: PLoS One. 2015 Aug 11;10(8):e0135538. doi: 10.1371/journal.pone.0135538 (PMC4532505; doi:10.1371/journal.pone.0135538)
Supplement: S1 Table — (DOCX) [file pone.0135538.s003.docx]

S1 Table. Accuracy assessment for the classification of Landsat images in 1993.

| Classified | Reference (Pixels) | | | | | | | | |
| --- | --- | --- | --- | --- | --- | --- | --- | --- | --- |
|  | MC | Sea | *S. alterniflora* | Mudflat | UL | OV | Total | UA(%) | F_1_ score |
| MC | 1355 | 259 | 0 | 128 | 43 | 0 | 1785 | 0.76 | 0.77 |
| Sea | 232 | 1802 | 0 | 181 | 6 | 0 | 2221 | 0.81 | 0.80 |
| *S. alterniflora* | 0 | 0 | 40 | 0 | 0 | 5 | 45 | 0.89 | 0.86 |
| Mudflat | 127 | 188 | 6 | 1354 | 41 | 71 | 1787 | 0.76 | 0.76 |
| UL | 16 | 0 | 0 | 82 | 486 | 22 | 606 | 0.80 | 0.82 |
| OV | 0 | 0 | 2 | 31 | 0 | 1182 | 1215 | 0.97 | 0.95 |
| Total | 1730 | 2249 | 48 | 1776 | 576 | 1280 | 7659 |  |  |
| PA(%) | 0.78 | 0.80 | 0.83 | 0.76 | 0.84 | 0.92 |  |  |  |

Overall accuracy = 81.2%.

Overall kappa statistics = 0.76

MC: Mudflat cultivation, UL: Urban land, OV: Other vegetation.
