# Supplementary material for: Monitoring the Invasion of Spartina alterniflora from 1993 to 2014 with Landsat TM and SPOT 6 Satellite Data in Yueqing Bay, China
Source: PLoS One. 2015 Aug 11;10(8):e0135538. doi: 10.1371/journal.pone.0135538 (PMC4532505; doi:10.1371/journal.pone.0135538)
Supplement: S2 Table — (DOCX) [file pone.0135538.s004.docx]

S2 Table. Accuracy assessment for the classification of Landsat images in 1996.

| Classified | Reference (Pixels) | | | | | | | | |
| --- | --- | --- | --- | --- | --- | --- | --- | --- | --- |
|  | MC | Sea | *S. alterniflora* | Mudflat | UL | OV | Total | UA(%) | F_1_ score |
| MC | 1493 | 407 | 0 | 273 | 51 | 0 | 2224 | 0.67 | 0.70 |
| Sea | 309 | 2002 | 0 | 351 | 5 | 0 | 2667 | 0.75 | 0.76 |
| *S. alterniflora* | 0 | 0 | 196 | 2 | 4 | 8 | 210 | 0.93 | 0.88 |
| Mudflat | 212 | 201 | 24 | 1994 | 53 | 83 | 2567 | 0.78 | 0.75 |
| UL | 21 | 0 | 0 | 42 | 603 | 55 | 721 | 0.84 | 0.84 |
| OV | 0 | 0 | 17 | 61 | 0 | 1556 | 1634 | 0.95 | 0.93 |
| Total | 2035 | 2610 | 237 | 2723 | 716 | 1702 | 10023 |  |  |
| PA(%) | 0.73 | 0.77 | 0.83 | 0.73 | 0.84 | 0.91 |  |  |  |

Overall accuracy = 78.3%.

Overall kappa statistics = 0.73.

MC: Mudflat cultivation, UL: Urban land, OV: Other vegetation.
