# Supplementary material for: Monitoring the Invasion of Spartina alterniflora from 1993 to 2014 with Landsat TM and SPOT 6 Satellite Data in Yueqing Bay, China
Source: PLoS One. 2015 Aug 11;10(8):e0135538. doi: 10.1371/journal.pone.0135538 (PMC4532505; doi:10.1371/journal.pone.0135538)
Supplement: S3 Table — (DOCX) [file pone.0135538.s005.docx]

S3 Table. Accuracy assessment for the classification of Landsat images in 1999.

| Classified | Reference (Pixels) | | | | | | | | |
| --- | --- | --- | --- | --- | --- | --- | --- | --- | --- |
|  | MC | Sea | *S. alterniflora* | Mudflat | UL | OV | Total | UA(%) | F_1_ score |
| MC | 1689 | 384 | 0 | 94 | 63 | 0 | 2230 | 0.76 | 0.77 |
| Sea | 322 | 2383 | 0 | 173 | 0 | 0 | 2878 | 0.83 | 0.82 |
| *S. alterniflora* | 0 | 0 | 404 | 5 | 2 | 53 | 464 | 0.87 | 0.85 |
| Mudflat | 139 | 165 | 26 | 1801 | 37 | 143 | 2311 | 0.78 | 0.81 |
| UL | 27 | 0 | 12 | 17 | 846 | 37 | 939 | 0.90 | 0.90 |
| OV | 0 | 0 | 49 | 22 | 0 | 1667 | 1738 | 0.96 | 0.92 |
| Total | 2177 | 2932 | 491 | 2112 | 948 | 1900 | 10560 |  |  |
| PA(%) | 0.78 | 0.81 | 0.82 | 0.85 | 0.89 | 0.88 |  |  |  |

Overall accuracy = 83.2%.

Overall kappa statistics = 0.79.

MC: Mudflat cultivation, UL: Urban land, OV: Other vegetation.
