# Supplementary material for: Monitoring the Invasion of Spartina alterniflora from 1993 to 2014 with Landsat TM and SPOT 6 Satellite Data in Yueqing Bay, China
Source: PLoS One. 2015 Aug 11;10(8):e0135538. doi: 10.1371/journal.pone.0135538 (PMC4532505; doi:10.1371/journal.pone.0135538)
Supplement: S4 Table — (DOCX) [file pone.0135538.s006.docx]

S4 Table. Accuracy assessment for the classification of Landsat images in 2003.

| Classified | Reference (Pixels) | | | | | | | | |
| --- | --- | --- | --- | --- | --- | --- | --- | --- | --- |
|  | MC | Sea | *S. alterniflora* | Mudflat | UL | OV | Total | UA(%) | F_1_ score |
| MC | 1764 | 538 | 55 | 366 | 63 | 0 | 2786 | 0.63 | 0.68 |
| Sea | 415 | 1983 | 0 | 143 | 0 | 0 | 2541 | 0.78 | 0.75 |
| *S. alterniflora* | 0 | 0 | 1572 | 11 | 43 | 143 | 1769 | 0.89 | 0.84 |
| Mudflat | 227 | 233 | 61 | 1642 | 186 | 205 | 2554 | 0.64 | 0.69 |
| UL | 28 | 8 | 92 | 121 | 795 | 41 | 1085 | 0.73 | 0.73 |
| OV | 0 | 0 | 187 | 32 | 0 | 1564 | 1783 | 0.88 | 0.84 |
| Total | 2434 | 2762 | 1967 | 2315 | 1087 | 1953 | 12518 |  |  |
| PA(%) | 0.72 | 0.72 | 0.80 | 0.71 | 0.73 | 0.80 |  |  |  |

Overall accuracy = 74.4%.

Overall kappa statistics = 0.69.

MC: Mudflat cultivation, UL: Urban land, OV: Other vegetation.
