# Supplementary material for: Monitoring the Invasion of Spartina alterniflora from 1993 to 2014 with Landsat TM and SPOT 6 Satellite Data in Yueqing Bay, China
Source: PLoS One. 2015 Aug 11;10(8):e0135538. doi: 10.1371/journal.pone.0135538 (PMC4532505; doi:10.1371/journal.pone.0135538)
Supplement: S5 Table — (DOCX) [file pone.0135538.s007.docx]

S5 Table. Accuracy assessment for the classification of Landsat images in 2006.

| Classified | Reference (Pixels) | | | | | | | | |
| --- | --- | --- | --- | --- | --- | --- | --- | --- | --- |
|  | MC | Sea | *S. alterniflora* | Mudflat | UL | OV | Total | UA(%) | F_1_ score |
| MC | 2490 | 272 | 0 | 220 | 68 | 0 | 3050 | 0.82 | 0.82 |
| Sea | 253 | 1922 | 0 | 280 | 0 | 0 | 2455 | 0.78 | 0.81 |
| *S. alterniflora* | 0 | 0 | 3450 | 0 | 7 | 177 | 3634 | 0.95 | 0.92 |
| Mudflat | 307 | 102 | 108 | 2222 | 45 | 77 | 2861 | 0.78 | 0.78 |
| UL | 5 | 0 | 22 | 85 | 735 | 57 | 904 | 0.81 | 0.84 |
| OV | 0 | 0 | 257 | 62 | 0 | 1754 | 2073 | 0.85 | 0.85 |
| Total | 3055 | 2296 | 3837 | 2869 | 855 | 2065 | 14977 |  |  |
| PA(%) | 0.82 | 0.84 | 0.90 | 0.77 | 0.86 | 0.85 |  |  |  |

Overall accuracy = 83.9%.

Overall kappa statistics = 0.80.

MC: Mudflat cultivation, UL: Urban land, OV: Other vegetation.
