# Supplementary material for: Monitoring the Invasion of Spartina alterniflora from 1993 to 2014 with Landsat TM and SPOT 6 Satellite Data in Yueqing Bay, China
Source: PLoS One. 2015 Aug 11;10(8):e0135538. doi: 10.1371/journal.pone.0135538 (PMC4532505; doi:10.1371/journal.pone.0135538)
Supplement: S6 Table — (DOCX) [file pone.0135538.s008.docx]

S6 Table. Accuracy assessment for the classification of Landsat images in 2009.

| Classified | Reference (Pixels) | | | | | | | | |
| --- | --- | --- | --- | --- | --- | --- | --- | --- | --- |
|  | MC | Sea | *S. alterniflora* | Mudflat | UL | OV | Total | UA(%) | F_1_ score |
| MC | 1822 | 376 | 0 | 96 | 59 | 0 | 2353 | 0.77 | 0.76 |
| Sea | 351 | 2213 | 0 | 263 | 0 | 0 | 2827 | 0.78 | 0.79 |
| *S. alterniflora* | 0 | 0 | 3689 | 8 | 39 | 183 | 3919 | 0.94 | 0.90 |
| Mudflat | 219 | 154 | 156 | 1787 | 86 | 113 | 2515 | 0.71 | 0.76 |
| UL | 31 | 0 | 102 | 21 | 827 | 32 | 1013 | 0.82 | 0.82 |
| OV | 0 | 0 | 287 | 32 | 0 | 1631 | 1950 | 0.84 | 0.83 |
| Total | 2423 | 2743 | 4234 | 2207 | 1011 | 1959 | 14577 |  |  |
| PA(%) | 0.75 | 0.81 | 0.87 | 0.77 | 0.82 | 0.83 |  |  |  |

Overall accuracy = 82.1%.

Overall kappa statistics = 0.78.

MC: Mudflat cultivation, UL: Urban land, OV: Other vegetation.
